# Factors Associated with Loneliness in Rural Older Adults during the COVID-19 Pandemic: Focusing on Connection with Others

**DOI:** 10.3390/healthcare10030484

**Published:** 2022-03-04

**Authors:** Hiyori Hanesaka, Michiyo Hirano

**Affiliations:** 1 Graduate School of Health Sciences, Hokkaido University, Sapporo 060-0812, Japan; comuharu1125@gmail.com; 2 Faculty of Health Sciences, Hokkaido University, Sapporo 060-0812, Japan

**Keywords:** COVID-19, older adults, rural, loneliness, neighborhood, independent view of self, interdependent view of self

## Abstract

The spread of COVID-19 is considered to have strengthened people’s awareness of others. Additionally, the COVID-19 pandemic has reduced connection with others among older adults and increased loneliness. This study aimed to investigate the factors affecting loneliness among older adults in rural areas during the COVID-19 pandemic by focusing on the connection with others. The target group included 932 rural Japanese adults, aged 65–74 years. An anonymous, self-administered questionnaire survey was conducted. Valid responses were obtained from 405 participants (valid response rate: 43.5%). A multiple regression analysis was performed using the forced entry method with loneliness as the dependent variable. The independent variables were those showing significant associations with loneliness based on the univariate analysis. Sex (β = −0.139), economic situation (β = −0.103), neighborhood ties (β = −0.260), independent view of self (β = −0.213), interdependent view of self (β = 0.171), and communication through phone (β = −0.128) were significantly associated with loneliness. Connection with others and subjective views of the relationship between self and others were associated with loneliness in situations where one was more aware of the behavior of oneself and others in an infectious disease pandemic.

## 1. Introduction

By 15 December 2021, COVID-19 had caused 270,031,622 infections and 5,310,502 deaths [1]. The number of people infected by COVID-19 has varied by country owing to varying waves of the infection. In particular, Japan has experienced five instances of a rapid increase in the number of infected people between February 2020 and October 2021 [2]. This frequency was higher than the rest of the world. According to the WHO, older people are at higher risk of experiencing more serious illness from COVID-19 [3]. COVID-19 is a threat to older people, especially in Japan, where the aggravation rate is 25 and 47 times higher for those in their 60s and 70s, respectively, compared to those in their 30s [4]. Therefore, the spread of the infection greatly affects older people in Japan.

To control the spread of COVID-19 infection, various infection prevention measures, including “lockdown,” were implemented worldwide. In Ireland, a fine was imposed for not wearing a mask [5], and in the United Kingdom, an outing-related fine was enforced [6]. Japan did not implement any legally binding compulsory measures. Rather, a state of emergency was issued and people were asked to cooperate in reducing the chances of unnecessary and unurgent outings and contact between people [7]. In other words, the actions of Japanese people regarding infection prevention were determined by free choice.

Although the compulsory measures taken by various countries vary, people’s social activities are decreasing due to the COVID-19 pandemic. A joint study by 35 research institutes in Asia, Africa, and Europe reported that COVID-19 has significantly reduced social activities with friends [8]. Based on this, opportunities for older adults to interact with others are decreasing due to the COVID-19 pandemic. 

Older adults have placed more importance on how their neighbors judge the risk of infection while deciding whether to carry out activities during the COVID-19 pandemic [9]. Therefore, consciousness, which can be defined as being conscious of others and caring about their evaluation, may be associated with the psychological state of older adults during the COVID-19 pandemic. The scale for measuring independent and interdependent views of the self which was proposed by Takata also indicates consciousness of others [10]. It indicates the degree to which one recognizes oneself as being separated from, or connected, to others. Psychological processes, such as cognition, emotion, and motivation differ greatly, depending on the degree of one’s independent and interdependent view of oneself [11]. Consciousness of others may be associated with the psychological state of older adults during the COVID-19 pandemic.

Recently, many studies have focused on the psychological effects of the COVID-19 pandemic on older people. Due to COVID-19, depressive symptoms, fatigue [12], and social isolation due to loss of social interaction [13] are increasing. In addition, loneliness in older people, which has a deep psychological impact, is also increasing [14]. Loneliness in older people is an important issue as their well-being is linked to physical health, mental health, and mortality [15]. 

Loneliness is defined as “the unpleasant experience that occurs when a person’s network of social relationships is deficient in some important way, either quantitatively or qualitatively” [16]. Currently, as the COVID-19 pandemic has reduced socializing with friends [8], some people may find it an unpleasant experience to be unable to socialize with others as they did before the COVID-19. Therefore, loneliness is considered an important problem as the COVID-19 pandemic has reduced socializing.

Gender, marital status, residential area [17], race, education and economic status [18] have been reported as factors of loneliness in older adults before the COVID-19 pandemic. After the COVID-19 pandemic, living alone, gender, having four or more chronic illnesses [19], and educational level [20] were factors of loneliness in older adults. In a study comparing the factors of loneliness before and after the COVID-19, economic conditions, race, gender, place of residence, and educational history were reported as factors of loneliness even before the COVID-19. However, after COVID-19, they became more strongly associated with loneliness [21]. 

Relief from loneliness, in older adults, is associated with neighborhood relationships [22], participation in group activities [23], and having multiple belonging groups [24]. The degree of interaction with others, the size of the social network [17], face-to-face and non-face-to-face contact with friends and relatives [25], and telephone interaction and frequency [26] are also associated with loneliness in older people. Various social relations, such as belonging groups and socializing with friends, are important factors in the loneliness levels of older adults. Therefore, it is necessary to investigate the factors associated with loneliness in older adults from the perspective of connection with others during the COVID-19 pandemic.

This study focused on older adults living in rural areas. Compared with urban areas, older adults in rural areas seek more intimate and informal connections, and visits from friends and neighbors enhance their subjective well-being [27]. Connection with others is considered a major health-related factor among older adults living in rural areas.

Therefore, this study aimed to examine the factors affecting loneliness among older adults in rural areas during the COVID-19 pandemic by focusing on their connection with others. Examining factors impacting loneliness would facilitate recommendations of lifestyle changes for older adults in rural areas, thus helping them maintain their mental health in a post-COVID-19 society.

## 2. Materials and Methods

### 2.1. Target Area

The target area was Town A in Hokkaido, Japan. The population of Town A is approximately 5100, with an aging rate of 43.1%. The first state of emergency in Japan lasted from 7 April to 31 May 2020, and the second state of emergency lasted from 7 January to 21 March 2021 [7]. In Hokkaido, where Town A is located, the first wave occurred in January 2020, the second occurred at the end of March 2020, and the third wave started at the end of October 2020, and continued until the end of February [28]. As of 14 December 2021, the total number of infected individuals in Hokkaido was 61,204. We conducted a survey when the number of infected people began to decrease, after the third wave.

### 2.2. Participants

The participants were 932 men and women aged 65–74 years living in Town A. In Japan, 65–74 years old is defined as young-old. For 65–74 years, the certification rate for requiring help is 1.4%, and the certification rate for long-term care is 2.9%. For people aged 75 years and above, the certification rate for requiring help is 8.8%, whereas that for long-term care is 23.0% [29]. The health status of older adults is significantly different between the young-old and old-old groups. The young-old are highly active because the effects of the restrictions imposed by the deterioration of their health conditions are minimal; therefore, it is possible for them to actively interact and connect with others. The target population was selected based on the above parameters.

### 2.3. Research Design

Quantitative and descriptive study were chosen as the research design.

### 2.4. Conceptual Framework

**Figure 1:**
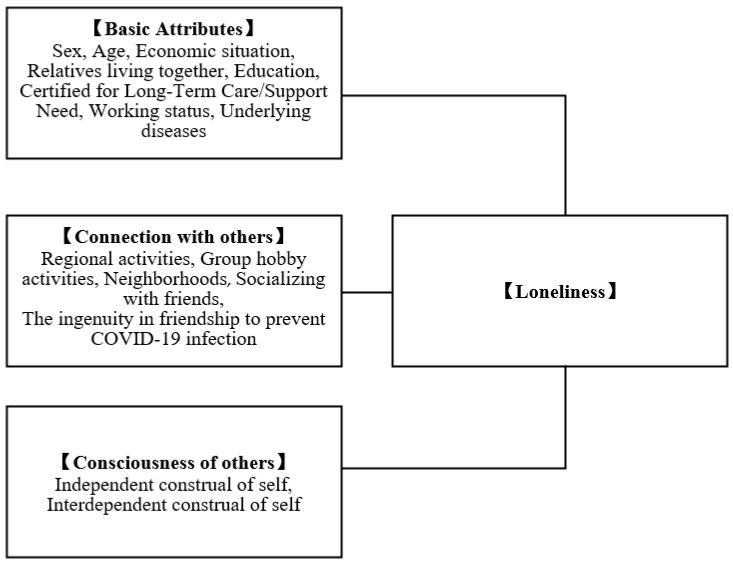
Conceptual framework.

The conceptual framework of this study is shown in Figure 1. In this study, loneliness was defined as “the unpleasant experience that occurs when a person’s network of social relationships is deficient in some important way, either quantitatively or qualitatively” [16]. In addition, connection with others was defined as “subjectively perceiving a social relationship with others” with reference to previous studies of loneliness [16]. In previous studies, belonging groups [24], such as neighborhood [22] and group activities [23], were associated with loneliness. Regarding relationships with friends, face-to-face and non-face-to-face methods [25] and frequency [26] were associated with loneliness. Therefore, we focused on the frequency and method of socializing with friends. From the above, “others” in this study includes friends and people related to the group to which the participants belonged, including members of neighborhoods and group activities.

The factors comprised basic attributes, connection with others, consciousness of others, and loneliness. Sex, age, economic situation, relatives living together, education, being certified for long-term care/requiring help, working status and underlying diseases were the factors comprising basic attributes. Regional activities, group hobby activities, neighborhoods, socializing with friends and the ingenuity in friendship to prevent COVID-19 infection were categorized under connection with others, whereas independent view of self and interdependent view of self comprised awareness of others. In this study, without setting adjustment variables, basic attributes, connection with others and awareness of others were treated equally, and all variables were treated as independent variables.

### 2.5. Survey Method

An anonymous, self-administered questionnaire survey was conducted by mail in March, 2021. See Table 1 for the attributes of the participants.

Based on the white paper on an aging society, we asked participants to choose from five alternatives: “almost no relationship,” “greetings,” “some relationship other than greetings,” and “intimate relationship” to understand connection with others in the neighborhood [29]. Respondents were asked about their participation in regional activities and group hobby activities. 

Regarding connection with others, in the method and frequency of socializing with friends, we informed the participants, “I will ask you about your friendships for the past year after the COVID-19 pandemic (from March 2020 to the present).” We divided the methods of socializing with friends into five categories. These included “talking directly with a small number of people at home or in a store,” “talking directly in a group,” “communicating through phone,” “communicating through email,” and “communicating through videophone (PC or smartphone).” We measured their frequency by asking them to pick from five alternatives: “nearly every day,” “several times per week,” “several times per month,” “several times per year,” and “do not.” To measure the ingenuity in friendships to prevent COVID-19, we informed the participants, “We will ask you about the ingenuity in friendship to prevent COVID-19 infection since the pandemic began.” As for the ingenuity, we referred to “About the Ministry of Health, Labour and Welfare’s Response to the New Coronavirus (For Older People)” [30] where the Ministry of Health, Labour and Welfare recommends “refraining from going out” as an infection control measure. Therefore, “reduced frequency of socializing with friends” was set as a question. Furthermore, sealing, crowding, and avoiding close contact were recommended. Therefore, “meeting after taking infection prevention measures such as masks, ventilation, and avoidance of congestion,” was set as a question. The same site recommends interacting with family and friends through letters, emails, SNS, etc. Therefore, “changed methods of contact from in-person contact to others” was set as a question. The participants could respond either “Yes” or “No.” Japanese people showed ingenuity in friendship to prevent COVID-19 infection by their own free choice. Since this study focused on connections with others, we selected ingenuity while meeting with others as following the infection control measures recommended by the Ministry of Health, Labor and Welfare.

The awareness of others was composed of independent and interdependent view of self. An independent view of self was defined as one in which one “sees oneself as a unique entity separated from others” [10], and the concepts included within it included “individual recognition/assertion” and “dogmatism.” Interdependent view of self was defined as “seeing oneself as part of a relationship that is connected to another” [10], and the concepts included in it were “affinity and adaptation to others” and “evaluation concerns.” We used the shortened version of the Takata scale to measure independent and interdependent view of self [10]. This scale consists of 10 items, with a score ranging from 1–7 points for both independent and interdependent view of self. A high score indicates a high degree of independent/interdependent view of self. 

The Japanese version of the UCLA Loneliness Scale (Version 3) [31] was used to measure loneliness. This scale consists of 20 items, with four reverse choices: never, almost never, sometimes, and always. The score ranges from 20 to 80 points; the higher the score, the stronger the feeling of loneliness. The definition of loneliness on this scale is the same as described above.

### 2.6. Analytical Method

To examine the factors affecting loneliness experienced by older adults during COVID-19, the relationships between loneliness and basic attributes, loneliness and connection with others, and loneliness and awareness of others were analyzed using *t*-tests and one-way ANOVAs. We performed a multiple regression analysis using the forced entry method with the items found to be related in the above-mentioned univariate analysis as the independent variables and loneliness as the dependent variable. The standardized partial regression coefficient (hereinafter referred to as β) and 95% confidence interval (hereinafter referred to as 95% CI) were then calculated. SPSS Statistics ver. 22 was used for the analysis, and the significance level was set at 5%.

## 3. Results

Of the 932 survey copies distributed, 415 were answered (recovery rate: 44.5%). The number of valid responses was 405, excluding 10 who had a defect in 10 or more responses (valid response rate: 43.5%).

### 3.1. Basic Attributes

Table 1 shows the characteristics of the participants. The participants included 205 men (50.6%) and 200 women (49.4%), with an average age of 69.69 ± 2.87 years. Regarding work status, 205 (50.6%) answered “yes” and 199 (49.1%) answered “no.” Out of 405 participants, 249 (61.5%) had underlying diseases.

### 3.2. Connection with Others

Table 2 shows connection with others.

In terms of regional activities, 217 (53.6%) participants reported “participating,” and 188 (46.4%) reported “not participating.” Regarding group hobby activities, 131 (32.3%) reported “participating,” and 269 (66.4%) reported “not participating.” Regarding neighborhood, 78 (19.3%) participants reported “intimate relationship,” 214 (52.8%) reported “some relationship other than greetings,” 97 (24.0%) reported “greetings” and 15 (3.7%) reported “almost no relationship.” Regarding the method of socializing with friends, “communicating through phone” was the most common option, with 11 (2.7%) participants communicating “almost every day,” 57 (14.1%) communicating “several times per week,” 151 (37.3%) communicating “several times per month,” and 110 (27.2%) communicating “several times per year,” while 76 (18.8%) did not communicate through phone with their friends. Regarding the ingenuity in friendship to prevent COVID-19 infection, 361 (89.1%) participants said they had “reduced frequency of socializing with friends,” 370 (91.4%) said they were “meeting after taking infection prevention measures such as masks, ventilation, and avoidance of congestion,” and 276 (68.1%) said they “changed methods of contact from in-person to others.”

### 3.3. Consciousness of Others

The average score of independent view of self was 4.31 ± 1.16 points, and the median was 4.38 points. The mode was 4.00 points for 67 (16.6%), the maximum value was 7.00 points for 11 (2.7%), and the minimum value was 1.00 points for 4 (1.0%). The average score of interdependent view of self was 4.10 ± 1.02 points, and the median was 4.25 points. The mode was 4.50 points for 45 (11.4%), the maximum value was 6.75 points for 2 (0.5%), and the minimum value was 1 point for 1 (0.2%).

### 3.4. Loneliness

The average score for loneliness using the Japanese version of the UCLA Loneliness Scale was 42.09 ± 9.29 points, with a median of 41 points. The mode was 41 points for 22 (5.6%), the maximum value was 79 points for 1 (0.3%), and the minimum value was 20 points for 1 (0.3%).

### 3.5. Factors Associated with Loneliness during COVID-19

Table 3 shows the results of the multiple regression analysis. When multicollinearity of the independent variables was confirmed, the variance inflation factors for all were found to be less than 2.0, and no multicollinearity was observed between the variables. As a result of the univariate analysis of loneliness with each variable, the items for which a significant difference was found were: for basic attributes, sex and economic situation; for connection with others, regional activities, group hobby activities, neighborhood, socializing with friends (talking directly with a small number of people at home or in a store, talking directly in a group, communicating through phone, communicating through email); and for consciousness of others, independent and interdependent view of self. The above items were input, using the forced entry method, as independent variables. The entire model was significant (*p* < 0.001), with an adjusted coefficient of determination of 0.275. As factors significantly related to loneliness, sex (β = −0.139, 95%CI: −4.366 to −0.802), economic situation (β = −0.103, 95%CI: −2.798~−0.140), neighborhood (β = −0.260, 95%CI: −4.451~−1.848), independent view of self (β = −0.213, 95%CI: −2.422~−0.966), interdependent view of self (β = 0.171, 95%CI: 0.754~2.346), and communicating through phone (with friends) (β = −0.128, 95%CI: −2.202~−0.121) were extracted.

## 4. Discussion

### 4.1. Factors Associated with Loneliness during COVID-19

To examine the factors affecting loneliness among older adults in rural areas during the COVID-19 pandemic by focusing on their connections with others, we conducted a survey of all young-old people living in Town A.

The average score of loneliness of the participants was 42.09 ± 9.29 points, while the loneliness score of community-dwelling older people in Japan before the COVID-19 pandemic was reported to be 42.2 ± 9.9 points [31]. Therefore, it can be considered that the loneliness of the participants was almost at the same level as that of Japanese older people in general. The loneliness of community-dwelling older adults in the United States and Turkey have been found to be 35.3 ± 7.6 points [26], and 40.5 ± 12.1 points [32], respectively. Therefore, it can be said that loneliness among older Japanese adults was slightly higher than that of other countries. 

In the basic attributes, sex and economic situation were related to loneliness. For connection with others, “communication through phone,” “socializing with friends,” and “neighborhood” were related to loneliness. In addition, in the awareness of others dimension, both an independent and interdependent view of self were related to loneliness. Regardless of the COVID-19 pandemic, sex and economic conditions are important factors associated with the loneliness of older adults [17,18,19,33]. In this study, sex and economic conditions were also associated with loneliness. Furthermore, they can be regarded as being important factors associated with loneliness even during the COVID-19 pandemic conditions. In addition, from the results of the multiple regression analysis, “neighborhood,” “communicating through phone” “interdependent view of self,” and “independent view of self” were the influencing factors that exceeded the standardized partial regression coefficient of economic conditions. Therefore, these four variables, under the categories of connection with others and awareness of others, impacted loneliness among older people during the COVID-19 pandemic. We discuss these four variables below.

First, we discuss the neighborhood in connection with others. For older adults, local non-kin ties are a much newer type of relationship than nonlocal non-kin ties [34]. Old age may entail changes in relationships due to certain factors, such as retirement, the deaths of friends and relatives, and movement closer to family. To address and supplement the loss associated with these life events and changes, older adults can build and maintain new local relationships. Those who have relationships with their neighbors have reported a low feeling of loneliness [22]. In Japan, the percentage of people who are “interacting” with their neighbors is reported to be 65.4%, while the percentage of those who are not interacting is increasing annually [35]. Age is factor in the degree of closeness with neighbors, as higher percentages of those who are “interacting” are in their 60s, 70s, and above [35]. Thus, it can bee seen that older adults are relatively close to each other, although relationships among neighbors in Japan are declining. Neighborhood relations are important for older people to maintain and reduce feelings of loneliness. 

Second, one of the aspects of connection with others, “communicating through phone” was the only significant factor associated with loneliness in the methods of socializing with friends. In addition, most of the participants in this study answered “communicating through phone” was their actual means of socializing with friends during COVID-19. Previous studies have found a significant association between the frequency of phone calls with friends and loneliness in older adults [26], and the results of this study support these findings. Even during the COVID-19 pandemic, it became clear that socializing with friends by “communicating through phone” played an important role in reducing loneliness. In addition, the largest number of participants selected this method of socializing, indicating that the telephone is an easy way for older adults to interact with friends. During COVID-19, older adults who reduced the frequency of going out had a significantly higher frequency of phone calls than those who did not reduce the frequency of going out [36]. In the present study, 89.1% of the participants said they had “reduced frequency of socializing with friends.” Based on the reduced rate of face-to-face interactions that involve going out, the COVID-19 pandemic may have increased the importance of non-face-to-face interactions. 

In Japan, to prevent COVID-19 infection, avoiding active face-to-face interaction with others was recommended. Under such circumstances, relationships with society and others may become diluted. The results of this study showed that “deep relationships” in the neighborhood and “telephone” as the method of interacting with friends were related to the reduction of loneliness. During COVID-19, the feelings of having deep relationships with others and the ability to have timely socialization in a non-face-to-face manner leads to a feeling of connection with others. It is presumed that older people subjectively perceived social relationships through such connections with others.

Generally, older adults live with various connections with others. The size of social networks is associated with loneliness in older adults [17]. Senior citizens’ social networks include informal networks, such as family, friends and neighbors, and formal networks, such as participants in the local healthcare system. In this study, considering that outings and face-to-face interaction were reduced during the pandemic, we did not investigate connections with various others shown via social networks. However, it was considered to be a localized but important result that connections with others in the neighborhood and communicating through phone with friends was found to be related to the loneliness of older adults during the pandemic.

Both the independent and interdependent view of self were associated with loneliness in older adults during the pandemic of COVID-19. There is a relationship between interdependence and loneliness [37]. The results of this study supported those of the previous study described above. An interdependent view of self consists of “affinity and adaptation to others (emphasis on cooperation and avoiding conflicts with others)” and “evaluation concerns (be aware of others and care about evaluation).” Although they are free to choose their own infection control measures, the COVID-19 epidemic has restricted people from being involved in social relations, such as by avoiding crowds and refraining from going out [30]. Prioritizing the eyes of others over their own intentions and socializing with others possibly affected the interdependent view of self and loneliness, as some people have a strong tendency to be conscious of others and to care about evaluation. 

Independent view of self consists of “individual recognition/assertion (Tendency to recognize oneself as a different being from others)” and “dogmatism (Act based on your own judgment without paying attention to others)” [10]. An independent view of self is positively related to “self-esteem” [38], and negatively related to “consideration for feelings toward others” [39] and “frequency of social comparisons in everyday situations” [40]. Therefore, it is considered that people with such a view act by giving priority to their own judgment rather than to what others think. Even if there were a lack of social relations due to the COVID-19 pandemic, it may not have led to an unpleasant experience because the behavior could be selected by one’s own judgment.

Such a causal relationship cannot be clarified in this study design. However, it is possible that independent/interdependent view of self are related to loneliness due to the restricted socialization with others during the COVID-19 pandemic and the environment in which participants could choose their own actions for infection control.

### 4.2. Practical Implications

Opportunities for face-to-face interactions among older adults have been forcibly reduced due to the COVID-19 pandemic [8]. In this study, it was found that about 90% of the respondents reduced their frequency of socializing with friends. The most common way to socialize with friends was found to be by phone. Those who have both face-to-face and non-face-to-face contact have been found to be less lonely than those who do not [25], and those who are able to interact face-to-face are more likely to engage in mobile communication, such as by telephone and email [41]. Thus, to reduce loneliness in the future, it is important to keep in touch with friends through both face-to-face and non-face-to-face interactions, and to continue the relationships with friends while paying attention to infection prevention. It has been reported that information and communication technology (ICT) interventions are effective in reducing loneliness in older people [42,43]. By developing an effective ICT program that older adults can easily use, loneliness emerging from an infectious disease epidemic in the future can be reduced.

Since neighborhood is an important factor in loneliness, it is considered necessary to make efforts to build and maintain connections in the neighborhood. Owing to the occurrence of child independence, loss due to death of partner or friend, and reduction of activity range due to health conditions, the social network of older adults is smaller than it is among youth [44]. As a result, the number of people who feel lonely or become socially isolated in old age increases [44]. Thus, these findings show that a neighborhood is effective at reducing loneliness in older adults even during the COVID-19 pandemic. Healthcare professionals in government agencies must continue to create opportunities for older adults to increase their involvement in the neighborhood on a regular basis, while paying attention to infection prevention measures.

Regarding awareness of others, it is necessary to support older adults by enlightening them so that they do not compare themselves with others more than necessary and so they can take correct measures for infection prevention. In addition, older adults are likely to have a lower chance of interacting directly with others around them due to refraining from going out, which might lead to negative emotions. Therefore, supporting older people in feeling directly connected to others around them by using ICT that enables video calls can be effective.

### 4.3. Limitations

This study has some limitations. First, the adjusted coefficient of determination was 0.275, and many factors other than the survey items, are associated with loneliness, so care must be taken in interpretation of these data. Second, since this was a cross-sectional study, and it was not possible to compare the association of the results to a time when COVID-19 was not prevalent, causal relationships loneliness and related factors, such as independent/interdependent view of self could not be established. In the future, research that includes a longitudinal perspective investigating changes in the actual status of relationships with friends and loneliness must be conducted as the vaccination rate increases. Third, the participants of this study were limited to older adults living in rural areas. Characteristics of urban areas, such as infection status and regional cohesiveness, differ from those rural areas, and therefore, the results of this study need to be interpreted accordingly.

## 5. Conclusions

The factors associated with loneliness in rural older adults during the COVID-19 pandemic were the basic attributes of sex and economic situation, connection with others through the neighborhood and communicating with friends through phone, and consciousness of others through independent and interdependent views of self. Since the neighborhood is a factor associated with loneliness in rural older adults during the COVID-19 pandemic, connection with others in the local area has become especially important during the pandemic. Socializing with friends, especially over the phone, was found to be related to loneliness during the COVID-19 pandemic. This could be due to the fact that the telephone was an easy method for older adults to use to interact with others, and that the COVID-19 pandemic reduced opportunities to connect with others through face-to-face contact and increased the demand for connection with others through non-face-to-face contact. Therefore, it is necessary for older adults to have a connection with others in the local area while paying attention to infection control. Regarding consciousness of others, socializing with others was restricted; however, participants were able to choose their own actions for infection control. This suggests that independent/interdependent view of self may be related to loneliness.

## Figures and Tables

**Table 1:** Characteristics of the participants.

(*N* = 405)		
		n (%)
Sex	Men	205 (50.6)
	Women	200 (49.4)
Age	65–69	189 (46.7)
	70–74	211 (52.1)
	Unanswered	5 (1.2)
Economic situation	Very poor	46 (11.4)
	Poor	231 (57.0)
	Good	120 (29.6)
	Very good	5 (1.2)
	Unanswered	3 (0.7)
Relatives living together	Living alone	64 (15.8)
	Living together with a spouse	236 (58.3)
	Living with children	79 (19.5)
	Others	26 (6.4)
Education	Junior high school	109 (26.9)
	High school	214 (52.8)
	Vocational school/Junior college	63 (15.6)
	University and above	19 (4.7)
Certified for Long-term care /Support need	Yes	9 (2.2)
	No	392 (96.8)
	Unanswered	4 (1.0)
Working status	Yes	205 (50.6)
	No	199 (49.1)
	Unanswered	1 (0.2)
Underlying diseases	Yes	249 (61.5)
	No	154 (38.0)
	Unanswered	2 (0.5)

**Table 2:** Connection with others.

(*N* = 405)		
		n (%)
Regional activities	Yes	217 (53.6)
	No	188 (46.4)
Group hobby activities	Yes	131 (32.3)
	No	269 (66.4)
	Unanswered	5 (1.2)
Neighborhood	Almost no relationship	15 (3.7)
	Greetings	97 (24.0)
	Some relationship other than greetings	214 (52.8)
	Intimate relationship	78 (19.3)
	Unanswered	1 (0.2)
Socializing with friends		
Talking directly with a small number of people at home or in a store	Almost everyday	15 (3.7)
	Several times a week	59 (14.6)
	Several times a month	130 (32.1)
	Several times a year	89 (22.0)
	Do not	109 (26.9)
	Unanswered	3 (0.7)
Talking directly in a group	Almost everyday	2 (0.5)
	Several times a week	22 (5.4)
	Several times a month	88 (21.7)
	Several times a year	84 (20.7)
	Do not	208 (51.4)
	Unanswered	1 (0.2)
Communicating through phone	Almost everyday	11 (2.7)
	Several times a week	57 (14.1)
	Several times a month	151 (37.3)
	Several times a year	110 (27.2)
	Do not	76 (18.8)
Communicating through email	Almost everyday	10 (2.5)
	Several times a week	46 (11.4)
	Several times a month	102 (25.2)
	Several times a year	56 (13.8)
	Do not	187 (46.2)
	Unanswered	4 (1.0)
Communicating through videophone (PC or smartphone)	Almost everyday	4 (1.0)
	Several times a week	9 (2.2)
	Several times a month	15 (3.7)
	Several times a year	17 (4.2)
	Do not	357 (88.1)
	Unanswered	3 (0.7)
The ingenuity in friendship to prevent COVID-19 infection		
Reduced frequency of socializing with friends	Yes	361 (89.1)
	No	43 (10.6)
	Unanswered	1 (0.2)
Meeting after taking infection prevention measures such as masks, ventilation, and avoidance of congestion	Yes	370 (91.4)
	No	34 (8.4)
	Unanswered	1 (0.2)
Changed methods of contact from in-person contact to others	Yes	276 (68.1)
	No	127 (31.4)
	Unanswered	2 (0.5)

**Table 3:** Factors associated with loneliness in older adults during the COVID-19 pandemic.

(*N* = 368)						
	Partial Regression Coefficient	Standard Partial Regression Coefficient (β)	95%CI		*P*	VIF
			Lower Bound	Upper Bound		
Sex	−2.584	−0.139	−4.366	−0.802	0.005	1.207
Economic situation	−1.469	−0.103	−2.798	−0.140	0.030	1.135
Underlying disease	1.177	0.062	−0.532	2.886	0.176	1.061
Religional activities	−0.202	−0.011	−2.125	1.722	0.837	1.401
Group hobby activities	−1.057	−0.054	−3.038	0.924	0.295	1.324
Neighborhood	−3.149	−0.260	−4.451	−1.848	<0.001	1.507
Talking directly with a small number of people	−0.508	−0.063	−1.344	0.328	0.233	1.392
Talking directly in a group setting	−0.696	−0.072	−1.742	0.349	0.191	1.532
Communicating through phone	−1.161	−0.128	−2.202	−0.121	0.029	1.717
Communicating through email	0.341	0.043	−0.482	1.165	0.416	1.403
Independent view of self	−1.694	−0.213	−2.422	−0.966	<0.001	1.097
Interdependent view of self	1.550	0.171	0.754	2.346	<0.001	1.015

Note: Multiple regression analysis (using the forced entry method) was used as the analysis method; adjusted R^2^ = 0.275.

## Data Availability

The data supporting the findings of this study are available from the corresponding author upon reasonable request.
